# Characterization of Flagella Produced by Clinical Strains of *Stenotrophomonas maltophilia*

**DOI:** 10.3201/eid0809.010535

**Published:** 2002-09

**Authors:** Doroti de Oliveira-Garcia, Monique Dall'Agnol, Mónica Rosales, Ana C.G.S. Azzuz, Marina B. Martinez, Jorge A. Girón

**Affiliations:** * Laboratório Clínico do Instituto Dante Pazzanese de Cardiologia, São Paulo, Brazil; † Departamento de Microbiologia do Instituto de Ciências Biomédicas da Universidade de São Paulo, São Paulo, Brazil; ‡ Benemerita Universidad Autónoma de Puebla, Puebla, México; § Departamento de Análises Clínicas da Faculdade de Ciências Farmacêuticas da Universidade de São Paulo, São Paulo, Brazil

**Keywords:** *Stenotrophomonas maltophilia*, flagella, opportunistic infections, adhesion

## Abstract

*Stenotrophomonas maltophilia* is an emerging nosocomial pathogen associated with opportunistic infections in patients with cystic fibrosis, cancer, and HIV. Adherence of this organism to abiotic surfaces such as medical implants and catheters represents a major risk for hospitalized patients. The adhesive surface factors involved in adherence of these bacteria are largely unknown, and their flagella have not yet been characterized biochemically and antigenically. We purified and characterized the flagella produced by *S. maltophilia* clinical strains. The flagella filaments are composed of a 38-kDa subunit, SM_FliC_, and analysis of its N-terminal amino acid sequence showed considerable sequence identity to the flagellins of *Serratia marcescens* (78.6%), *Escherichia coli*, *Proteus mirabilis*, *Shigella sonnei* (71.4%), and *Pseudomonas aeruginosa* (57.2%). Ultrastructural analysis by scanning electron microscopy of bacteria adhering to plastic showed flagellalike structures within the bacterial clusters, suggesting that flagella are produced as the bacteria spread on the abiotic surface.

*Stenotrophomonas* (formerly *Pseudomonas* and *Xanthomonas*) *maltophilia* is a widespread environmental microorganism that has become an important opportunistic pathogen associated with nosocomial colonization and infection (1–7). These organisms have been recovered from water faucets, water traps, respirometers, sinks, suction catheters, and occasionally, from cultures of the hands of hospital personnel (5,8). Infection and colonization of implantable medical devices such as catheters and intravenous cannulae represent a major risk for hospitalized patients. *S. maltophilia* can cause septicemia, endocarditis, conjunctivitis, mastoiditis, meningitis, postoperative wounds, abscesses, urinary tract infections, and pneumonia (6,9–11). The isolation rates of *S. maltophilia* from the respiratory tracts of patients with cystic fibrosis and from cancer and HIV-infected patients with opportunistic infections is increasing (4,12,13). Adhesion of these bacteria to abiotic surfaces such as those of medical implants and catheters suggests the development of a biofilm that protects bacteria from natural immune defenses or from the action of antimicrobial compounds. Biofilms are made up of a community of bacteria immobilized and embedded in an organic polymer matrix composed of polysaccharides and proteins of bacterial origin (14–16). Management of infection and successful clinical outcome by means of antimicrobial therapy are complicated by the intrinsic resistance of the bacteria to multiple antimicrobial agents, including carbapenems, and to the natural protection that biofilms confer to the enclosed bacteria (8,14). Besides the ability to adhere to plastic, to survive and multiply within total parenteral nutrition and other types of intravenous infusions, and to produce extracellular enzymes (4,8), little information is available regarding virulence factors associated with the pathogenesis of these bacteria. Production of a protease and elastase appears to be important in the pathogenesis of *S. maltophilia–*associated infections (5,17).

While for some bacteria the expression of flagella does not clearly relate to pathogenesis, for a variety of bacterial pathogens, such as *Proteus mirabilis*, *Salmonella enterica*, and *Yersinia enterocolitica,* the participation of flagella in adherence and invasion has been documented (18–20). In addition, the role of flagella in the formation and development of biofilm has recently been investigated in *Pseudomonas, E. coli*, and *Vibrio cholerae* (21–24). Jucker et al. reported that nonspecific adhesion and biofilm formation by *S. maltophilia* to glass and Teflon may be attributed to the net positive surface charge of the bacteria (23). As with a variety of microorganisms, other surface determinants may confer the adhesive attributes necessary for *S. maltophilia-*specific adhesion. Although biofilm formation by *S. maltophilia* has been documented, no surface molecule or structure such as flagella or fimbrial adhesins implicated in adherence to plastic or eukaryotic cells has yet been characterized in detail (4,23,25). To characterize the surface appendages produced by *S. maltophilia*, we purified flagella from a clinical isolate and used specific anti-flagella antibodies to test for the presence of these structures in a collection of clinical isolates. In addition, we studied the kinetics of adhesion and performed ultrastructural studies by scanning electron microscopy of bacteria adhering to plastic. These studies showed structures resembling flagella, suggesting that these structures may be important for the adherence phenomenon.

## Materials and Methods

### Bacterial Strains and Growth Conditions

We included in this study 46 clinical isolates of *S. maltophilia* obtained from patients admitted to four institutions in the City of São Paulo, Brazil: Instituto Dante Pazzanese de Cardiologia, Hospital das Clínicas, Laboratório Fleury, and Hospital Universitário (Universidade de São Paulo). Most of these clinical strains were isolated from respiratory tract secretions obtained from intubated patients with pneumonia; in most cases, *S. maltophilia* was the only infectious agent found (25). *S. maltophilia* ATCC 13637 is a reference strain also used in our studies. For expression of flagella, bacteria were grown on trypticase soy agar supplemented with 5% defibrinated sheep blood (Oxoid, Basingstoke, England) at 37°C for 48 h.

### Transmission and Scanning Electron Microscopy

We analyzed the presence of flagella by negative staining and transmission electron microscopy. Bacteria were negatively stained for 2 min with 1% phosphotungstic acid (pH 7.4) on carbon-Formvar (Electron Microscopy Sciences, Fort Washington, PA) copper grids as previously described (26,27). For ultrastructural analysis, bacterial specimens were fixed in 2% formalin and processed for scanning electron microscopy. Briefly, glass or plastic coverslips containing the adherent bacteria were postfixed with 1% osmium tetraoxide, dehydrated by sequential ethanol concentrations, dried to critical point, and coated with a mixture of gold and paladium (27). The specimens were examined in a high-resolution Hitachi (Tokyo, Japan) scanning electron microscope.

### Isolation of Flagella

For purification of flagella, clinical isolate *S. maltophilia* SMDP92 was grown on 100 blood agar plates and harvested in 100 mL of 10 mM phosphate-buffered saline (PBS), pH 7.4. The flagella were detached from the bacterial cells by vigorous shaking, and the supernatant containing the sheared flagella was separated by centrifugation at 8,000 x *g* for 30 min (26). The flagella were separated from outer membrane proteins and other contaminants by precipitation with 60% saturation of ammonium sulfate for 18 h at 4°C. After centrifugation at 12,000 x *g* for 30 min, the flagella were resuspended in PBS, and insoluble contaminants were removed by a similar centrifugation step. The supernatant was subjected to a second cycle of 20% ammonium sulfate precipitation (26). After dialysis with distilled water to remove excess salts, the purity of the preparations was monitored by sodium dodecyl-sulfate polyacrylamide gel electrophoresis (SDS-PAGE) (28) and electron microscopy (26).

### Western Blotting and N-Terminal Amino Acid Sequence Analysis

For SDS-PAGE and Western blot, whole bacterial cell extracts or flagellar extracts were denatured and separated in 14% polyacrylamide gels and transferred onto polyvinylidene difluoride (PVDF) membranes (Millipore Corp., Bedford, MA) (27). The blot was reacted with anti-flagella antibodies and secondary anti-rabbit immunoglobulin (Ig) G conjugated to horseradish peroxidase (Sigma Chemical Co., St. Louis, MO). The reaction was developed with a mixture of diamino benzidine and 30% hydrogen peroxide (Sigma). A 38-kDa protein band of interest was excised from the PVDF membrane and subjected to N-terminal amino acid sequence analysis at the Instituto de Química, Universidade de São Paulo. Sequence analysis and homology studies with published flagellin sequences were performed by using the EMBL/GenBank (BLAST of National Center for Biotechnology Information, Bethesda,MD) software.

### Anti-Flagella Antibodies

Antibodies against *S. maltophilia* flagella were raised by immunization of New Zealand rabbits with the flagellin protein (38-kDa band) excised from Coomassie-blue stained gels. The bands were dried and homogenized in complete Freund’s adjuvant for the first dose and in incomplete adjuvant for the subsequent three weekly doses. Blood was collected at each immunization, and the presence of antibodies was monitored by Western blot. Antibodies against flagella obtained from *E. coli* E2348/69 (O127:H6), *Shigella flexneri,* and *S. sonnei* were available from previous studies (26,27,29).

### Adhesion to Inert Surfaces

Adhesion to abiotic surfaces was studied at different times by using polystyrene 24-well plates (Nunc, Naperville, IL) with or without glass coverslips (21). Twenty microliters of an overnight culture of bacteria grown in Luria-Bertani broth was added to the wells containing 1 mL of Dulbecco’s minimal essential medium (D-MEM), supplemented with high glucose, and incubated for 15 and 30 min and 1, 2, 3, 4, 5, 6, 18, 48, and 72 h. After three washes with PBS, the bound bacteria were fixed with methanol, stained with crystal violet, and visualized under a light microscope. Replica samples were fixed with 2% formalin for scanning electron microscopy as described. All experiments were conducted in triplicate and were repeated at least three times.

To quantitate bacterial adherence over time (from 30 min to 72 h), we performed an adherence assay in 96-well plates as described and measured the uptake of crystal violet staining by reading optical density at 620 nm (22).

## Results

### Characteristics of *S. maltophilia* Flagella

**Figure 1:**
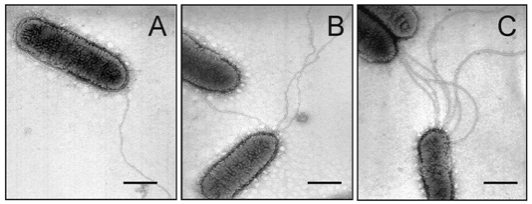
Electron micrographs showing expression of flagella by SMDP92. *Stenotrophomonas maltophilia* strains can have one (A) to several flagella (B,C). The flagella on these bacteria show a polar disposition. Bars, 0.5 µm

**Figure 2:**
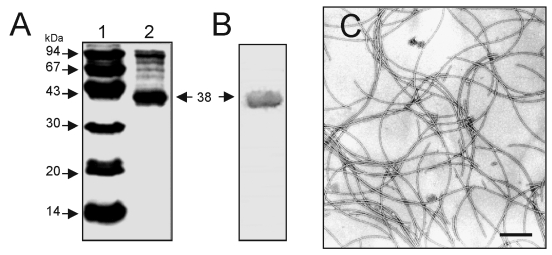
Analysis of flagella purified from SMDP92. (A) sodium dodecyl-sulfate polyacrylamide gel electrophoresis of purified flagella, showing the 38-kDa flagellin subunit. Lane 1, molecular weight standards; lane 2, purified SM_FliC_. (B) Immunoblotting and reactivity of purified flagella with anti-SM_FliC_ antibodies. The 38-kDa flagellin is indicated by an arrow. (C) Electron microscopy of purified flagella visualized by negative staining. Bar, 0.37 μm.

Growth of the bacteria in blood agar plates at 37°C resulted in a condition favorable for flagella expression. Analysis by electron microscopy demonstrated that while some organisms had only one polar flagellum, others had several flagellar structures (Figure 1). The flagella filaments, ~45 nm in width and >15 µm long, are indistinguishable from other unsheathed flagella such as those produced by *E. coli* or *Salmonella* (30,31). After purification of flagella by repeated ammonium sulfate precipitations, a peptide band that migrated with an apparent mass of 38 kDa was visualized in SDS-PAGE Coomasie blue-stained gels (Figure 2A). Antibodies obtained against the excised 38-kDa putative flagellin reacted with this polypeptide in immunoblots (Figure 2B). The flagella preparation was rich in flagellar filaments as determined by negative staining and electron microscopy (Figure 2C).

### Sequence and Antigenic Relatedness of *S. maltophilia* Flagellin to Other Flagellins

**Figure 3:**
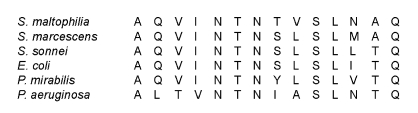
N-terminal amino acid sequence analysis. The first 14 residues of SM_FliC_ showed considerable identity to other flagellins. The highest degree of identity was found with the *Serratia marcescens* flagellin (78.6%). *Stenotrophomonas maltophilia* flagellin also showed identity to flagellins of *Shigella sonnei, Escherichia coli,* and *Proteus mirabilis* (71.4%), and the lowest identity was found with *Pseudomonas aeruginosa* flagellin (57.2%). The numbers on top indicate the amino acid position.

These results suggested that the 38 kDa is the major structural component (FliC) of the flagella filament. Thus, this polypeptide was subjected to N-terminal amino acid sequence analysis, which showed that the 38-kDa protein is in fact the flagellin structural protein, which is highly homologous to other bacterial flagellins. The *S. maltophilia* FliC protein, SM_FliC_, showed considerable identity in its first 14 amino acid residues to the flagellins of *E. coli*, *P. mirabilis*, and *Shigella sonnei* (71.4%), and 78.6% identity to the flagellin of *Serratia marcescens*. The flagella produced by *P. aeruginosa* showed the lowest level of identity (57.2%) with SM_FliC_ (Figure 3).

**Figure 4:**
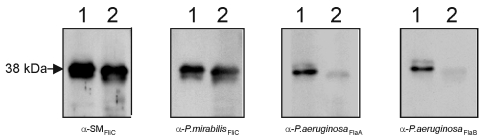
Reactivity of *Stenotrophomonas maltophilia* flagellin with different antibodies. Lane 1, SMDP92 strain; lane 2, ATCC 13637. Blots containing whole cells extracts of SMDP92 and ATCC 13637 were reacted with antibodies against SM_FliC_, flagella of *Proteus mirabilis,* and anti-FlaA and anti-FlaB of *Pseudomonas aeruginosa*.

Because of these sequence similarities, we were then interested in determining if SM_FliC_ shared any common epitopes with the other flagellins. This antigenic cross-reactivity was investigated by using several antisera against flagellins of *E. coli*, *Shigella,* *P. aeruginosa*, and *P. mirabilis*. Among these, only antibodies against flagella of *P. mirabilis* and anti-FlaA and anti-FlaB of *P. aeruginosa* reacted in immunoblots with the *Stenotrophomonas maltophilia* flagellins, although to differing levels of reaction (Figure 4). Anti-SM_FliC_ antibodies reacted with the SM_FliC_ proteins produced by both *S. maltophilia* strains tested (Figure 4A).

### Expression of Flagella by Clinical *S. maltophilia* Isolates

**Figure 5:**
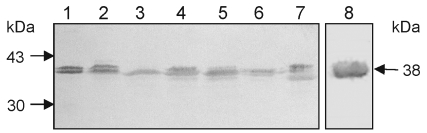
Identification of the 38-kDa flagellin protein SM_FliC_ in clinical isolates of *Stenotrophomonas maltophilia.* Lane 1, SMDP14; lane 2, SMDP275; lane 3, SMHC176; lane 4, SMHC181; lane 5, SMDP315; lane 6, SMDP314; and lane 7, SMHC179. Lane 8, the purified SM_FliC_, was used as positive control. The immunoblot shows the presence of the 38-kDa flagellin protein in all the isolates. Doublet bands were seen in some of the isolates. Molecular weight standards and the 38-kDa flagellin protein are indicated by arrows.

We investigated SM_FliC_ in fresh isolates of *S. maltophilia*. Forty-six *S. maltophilia* clinical isolates and *S. maltophilia* ATCC 13637 were studied by immunoblot, with antibodies against SM_FliC_ of SMDP92. A preparation of purified flagella was used in all reactions as a positive control. All the isolates tested produced the ~38-kDa flagellin that reacted with antibodies against SM_FliC_ (Figure 5). However, the molecular mass of the flagellin produced by some of the isolates differed slightly, and doublet bands were seen in some cases. We also performed negative staining and transmission electron microscopy in these isolates to confirm expression of flagella (Figure 1). These results show that the production of the 38-kDa flagellin and flagella is a common feature of reference and fresh clinical isolates of *S. maltophilia*.

### Kinetics of Adherence to Plastic

**Figure 6:**
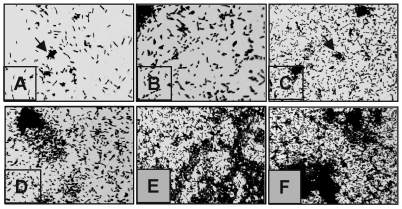
Kinetics of *Stenotrophomonas maltophilia* adherence to plastic. (A) As early as 30 min, individual bacteria have attached to the plastic surface and formed small clumps (arrows). (B–D) As incubation time proceeds for 1 (B), 2 (C), and 4 (D) h, the number of attached bacteria increases throughout the abiotic surface. (E) At 6 h, the bacterial monolayers progress into three-dimensional microcolonies (arrows). (F) After 18 h, the microcolonies have formed true bacterial communities. No obvious differences were noted beyond this incubation period. Magnification 400x.

**Figure 7:**
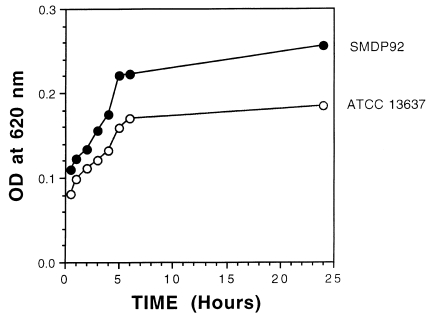
Graph showing kinetics of adherence by SMPD92 and ATCC 13637. Bacteria were allowed to bind to the plastic for 72 h and then were stained with crystal violet. Bacterial uptake of the dye was measured at 620 nm. Closed and open circles represent SMDP92 and ATCC13637, respectively.

As early as 30 min, individual bacteria were seen attaching to the glass surface and forming small clumps (Figure 6A). As the incubation time extended to 1, 2, and 4 h, the number of attached bacteria increased throughout the abiotic surface (Figure 6B–D). At 6 h, the adhering bacterial monolayer progressed into three-dimensional bacterial clumps (Figure 6E). After 18 h, extended areas of the glass surface were covered with large bacterial clumps (Figure 6F). No obvious differences were observed at incubation periods >18 h of infection (data not shown). The kinetics of adhesion were also monitored by quantification of crystal violet–stained-bacteria adhering to the 96-well plates. In correlation with the light microscopy micrographs shown above (Figure 6), a time-dependent adherence profile was obtained that reached a maximum level at 18 h (Figure 7), with no substantial increase in adherence beyond this period (data not shown).

**Figure 8:**
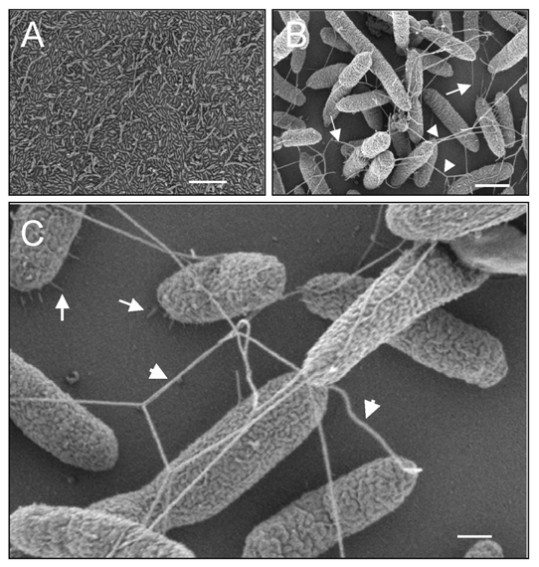
Ultrastructural analysis of *Stenotrophomonas maltophilia* adhering to plastic. (A) Scanning electron micrographs showing the tight adhesion of SMDP92 to the plastic surface. (B) Structures resembling flagella seem to be protruding and interconnecting bacteria (arrowheads) or connecting bacteria to the plastic (arrows). (C) In addition to the flagellalike filaments (arrowheads), high-power magnification shows the presence of thin fibrillar structures connecting bacteria to the abiotic surface. Bars: A 10 μm, B 1 μm, C 2 μm.

Furthermore, analysis by scanning electron microscopy of SMDP92 adhering to the plastic showed structures resembling flagella on the adhering bacteria (Figure 8). These filaments were seen protruding from the bacteria, apparently forming physical bridges between them. Thus, these filaments may play some yet-undefined role in adherence to plastic. High-power magnification of adhering bacteria showed flagella-like filaments (40–50 µm in width) and thin fibrillar structures (5–7 µm in width) resembling pili interconnecting bacteria and mediating adhesion of the bacteria to the abiotic surface (Figure 8).

## Discussion

Although adherence to abiotic surfaces is a property of both environmental and clinical *S. maltophilia* isolates, little information has been available to elucidate the nature of the surface factors involved in this phenomenon. Flagella have been associated with biofilm formation in other bacteria (18,20–22), where they can perform three basic roles: a) act as an adhesin promoting intimate attachment to the surface; b) generate force to subjugate the repulsive forces between bacteria and surface; and c) promote spread of the bacteria throughout the surface (20). In 1983 Montie and Stover purified flagella from several pseudomonads, including *P. maltophilia* strain B69 (now referred to as *Stenotrophomonas maltophilia*) (32), and found that B69 produced a flagellin and had a molecular mass of 33 kDa. They found that antisera against flagella of *P. aeruginosa* and *P. cepacia* did not agglutinate *P. maltophilia* bacteria, suggesting absence of antigenic cross-reactivity between these flagella. No further biochemical characterization of *S. maltophilia* flagella has been done. In this paper, we describe the purification and characterization of *S. maltophilia* flagella; we raised specific antibodies to study the production of flagella in a collection of clinical isolates. The flagella produced by *S. maltophilia* strains are composed of a 38-kDa flagellin subunit, SM_FliC_. The identity of this polypeptide was demonstrated by N-terminal amino acid sequencing analysis and by immunodetection assays using antibodies raised against the purified flagellin. The discrepancy between the molecular mass of SM_FliC_ and the flagellin (33 kDa) found previously in B69 could be attributed to differences in the electrophoresis conditions and molecular mass standards used, as well as to differences in the strains per se. Nevertheless, we did find molecular mass differences among flagellins produced by clinical isolates.

The comparison between the N-terminal amino acid sequence obtained from this 38-kDa polypeptide (14 residues) showed that SM_FliC_ shares important identity with several known flagellins: 71.4% identity to FliC of *E. coli*, *P. mirabilis*, and *S. sonnei*, and 78.6% identity to FliC of *Serratia marcescens*. *Stenotrophomonas* was previously considered a pseudomonad (2,3); however, the identity between the FliC of *S. maltophilia* and *P. aeruginosa* was 57.2%, which is lower than that observed with enterobacterial flagellins. In spite of the similarity between SM_FliC_ and these other flagellins, they are antigenically distinct since only antibodies against *P. mirabilis* flagellin, FlaA and FlaB of *P. aeruginosa* reacted with SM_FliC_ in immunoblots. We do not yet know the biological relevance of this finding, but based on these data, we can speculate that the flagellin gene of *S. maltophilia* was probably modified through the evolution of the organism, yielding a FliC protein with different antigenic properties but similar biological functions.

Visualization by high-resolution scanning electron microscopy of bacterial monolayers adhering to plastic showed flagellalike filaments connecting bacteria to each other and to the inert surface, suggesting that these structures are involved in adherence, along with other thin fibers, resembling pili. In *P. aeruginosa*, the flagella appear to act as structures that promote the initial interaction of the bacteria with the abiotic surface during early stages of biofilm development, as demonstrated with flagella mutants that are unable to produce biofilm (18). While the definitive role for flagella in adherence by *S. maltophilia* needs to be supported by the use of defined motility-lacking and flagella-deficient constructs, the presence of flagella at late stages of adherence on bacteria adhering to the plastic suggests that flagella may play some role in this event.

Much remains to be understood concerning the virulence mechanisms of *S. maltophilia.* The adherence of these bacteria to plastic may be important for the establishment of opportunistic infections in hospitalized and immunocompromised patients. Elucidating the surface factors that allow *S. maltophilia* to adhere to inert surfaces will contribute to the development of effective antimicrobial strategies for controlling these infections.
